# Supplementary material for: The behavioural and cognitive impacts of digital educational interventions in the emergency department: A systematic review
Source: PLOS Digit Health. 2025 Mar 26;4(3):e0000772. doi: 10.1371/journal.pdig.0000772 (PMC11942422; doi:10.1371/journal.pdig.0000772)
Supplement: S1 Appendix — (DOCX) [file pdig.0000772.s001.docx]

**S1 Appendix. Search strategies for this systematic review.**

| **Database** | **Search String** |
| --- | --- |
| Scopus | 1. ( "digital education" , AND hospital, "waiting room" ) 2. ( *"*education intervention"  *,*  AND “waiting room” ) 3. ( education,  AND hospital,  "education intervention"  ,  AND digital,  AND family ) 4. ( "education intervention"  ,  AND “waiting room” ) 5. ( "education intervention"  OR  "waiting room"  OR  digital ) 6. ( patient  AND education,  AND “waiting room” ) 7. ( "patient education"  ,  "waiting room" ) 8. ( "digital education"  ,  AND hospital ) 9. ( "digital education"  ,  AND hospital,  AND patient ) |
| Ovid MEDLINE | 1. Health education.mp. or exp Health Education 2. Mobile Applications.mp. or exp Mobile Applications 3. exp Patient Education as Topic 4. Pediatric Emergency Medicine.mp. or Pediatric Emergency Medicine 5. Emergency Service, Hospital.mp. or Emergency Service, Hospital/ or Emergency Medical Services 6. Digital Education or Intervention or Education or Computer-assisted instruction 7. exp Health Literacy/ec, es, st, td [Economics, Ethics, Standards, Trends] 8. 4 or 5 9. Patient.mp. 10. 7 and 9 11. 2 or 3 or 7 12. 8 and 11 |
| Cochrane | 1. MeSH descriptor: [Health Education] explode all trees 2. MeSH descriptor: [Mobile Applications] explode all trees 3. MeSH descriptor: [Patient Education as Topic] explode all trees 4. 1 or 2 or 3 5. MeSH descriptor: [Pediatric Emergency Medicine] explode all trees 6. MeSH descriptor: [Emergency Service, Hospital] explode all trees 7. 5 or 6 8. 4 and 7 |
