# Supplementary material for: The behavioural and cognitive impacts of digital educational interventions in the emergency department: A systematic review
Source: PLOS Digit Health. 2025 Mar 26;4(3):e0000772. doi: 10.1371/journal.pdig.0000772 (PMC11942422; doi:10.1371/journal.pdig.0000772)
Supplement: S2 Appendix — (DOCX) [file pdig.0000772.s002.docx]

**S2 Appendix. Original Protocol for this systematic review.**

**Review Title:** Systematic review of the behavioural and cognitive impacts of digital education interventions in the emergency department

**Anticipated Start Date:** 19/05/2022

**Anticipated Completion Date:** 31/03/2024

**Named Contact, Email:** Sophie Cleff, sophie.cleff@mail.mcgill.ca

**Organizational Affiliation:** Montreal Children’s Hospital

**Review Team Members:** Sophie Cleff, Shubhang Sreeranga, Jennifer Turnbull, Esli Osmanlliu

**Conflicts of Interest:** None

**Review Question:** 1. How do Emergency Department digital educational interventions impact caregivers and patients (based on behaviour, cognition, healthcare utilisation, clinical status, patient experience, and patient satisfaction)?

2. What are the characteristics of a successful educational intervention, and how is success measured?

**Searches:** No restriction on database, language, or time of publication.

**Condition or domain being studied:** Digital educational interventions and their impact on patient and caregiver health-related outcomes.

**Participants/population:** Included: Caregivers and patients who come through a hospital emergency department. Excluded: Physicians

**Interventions:** Included: Digital educational interventions given individually or as an adjunct to regular care or to analog methods. These interventions include educational videos, online tools, gamified educational material, and app or mobile-device based information. Excluded: Analog interventions only (pamphlets, interviews, posters)

**Comparator**: Included: Usual care (as control) or analog interventions (as head-to-head comparison) or pre-post comparisons. Excluded: Studies without controls or comparator

**Types of study to be included**: Only Randomized Control Trials

**Context**: Hospital emergency departments; before, during or after care

**Main outcome**: The primary outcome will be the impact of digital educational interventions on ED use and on behavioral & cognitive outcomes.

*Measures of effect:* Measures of behavioral outcomes will include changes in health literacy and changes related to care-seeking behavior. Cognitive outcomes will be measured by self-reported confidence with disease management, self-reported concerns about specific reasons for presentation, health literacy, motivation to make behavioral changes, disease awareness and understanding.

**Additional outcomes**: Additional outcomes include the impact on clinical status of the patient, patient & family experience and satisfaction.

*Measures of effect*: Differences in satisfactions between intervention and control groups. The effect of the intervention was measured by absolute difference with corresponding measure of precision.

**Data extraction (Selection and Coding)**: Studies will be included if all the criteria of the PICO are met. Namely, the population must be caregivers and/or patients who come through a hospital emergency department. The intervention must be a digital educational intervention given individually or as an adjunct to standard/methods. A digital educational intervention is defined in the WHO's third global survey on eHealth as the "use of information and communication technologies in support of health services" (WHO 2016). We use this definition of digital intervention in our inclusion criteria, with the additional specificity of the included interventions being patient-facing and educational in nature. There must be a comparison group that consists of usual/standard care or analog educational interventions. We are including RCTs for primary analysis and quasi-experimental studies for support in the discussion. The intervention must have taken place at an emergency department (hospital or community-based) before, during or after care. Finally, the primary outcome we are looking for is the impact of digital educational interventions on ED use and cognitive and behavioral outcomes.

Two blinded student researchers will screen the articles and decide to include or exclude based on title & abstract. Then a senior researcher will break ties and resolve conflicts. The studies that have been included will then be reviewed again based on the full text of the article through the same process. The two student reviewers will read the full text of articles and record relevant data about population, intervention, comparators, and outcomes in an Excel spreadsheet. Any conflicts about inclusion/exclusion based on the full text will again be resolved by a senior researcher. If there is any missing data, we will contact the corresponding authors for information. We will be using the Rayyan software to record decisions.

**Risk of bias assessment**: We will be assessing the methods of randomization, blinding, treatment allocation, drop-out rates and outcome measures. The Cochrane Risk of Bias tool for Randomized Controlled Trials (RoB2) will be used. The assessment will be conducted by two reviewers. Any disagreements between reviewers' judgement will be discussed and a third researcher will help with the resolution.

**Strategy for data synthesis**: Due to heterogeneity in the methods and study characteristics of the included studies, a meta-analysis is most likely unfeasible. Analysis of studies with quantitative data and qualitative data will be done separately.

**Analysis of subgroups or subsets**: Pediatric vs adults

**Type and method of review**: Systematic review of interventions

*Health area of review*: Child health, care of elderly, education, cardiovascular, respiratory disorders

**Language**: Any

**Country**: Any

**Dissemination plans**: Yes we plan to publish review on completion

**Keywords**: digital education; education intervention; patient education; health literacy
