# Supplementary figures and images for: The behavioural and cognitive impacts of digital educational interventions in the emergency department: A systematic review
Source: PLOS Digit Health. 2025 Mar 26;4(3):e0000772. doi: 10.1371/journal.pdig.0000772 (PMC11942422; doi:10.1371/journal.pdig.0000772)

**S3 Appendix. PRISMA checklist for this systematic review.**


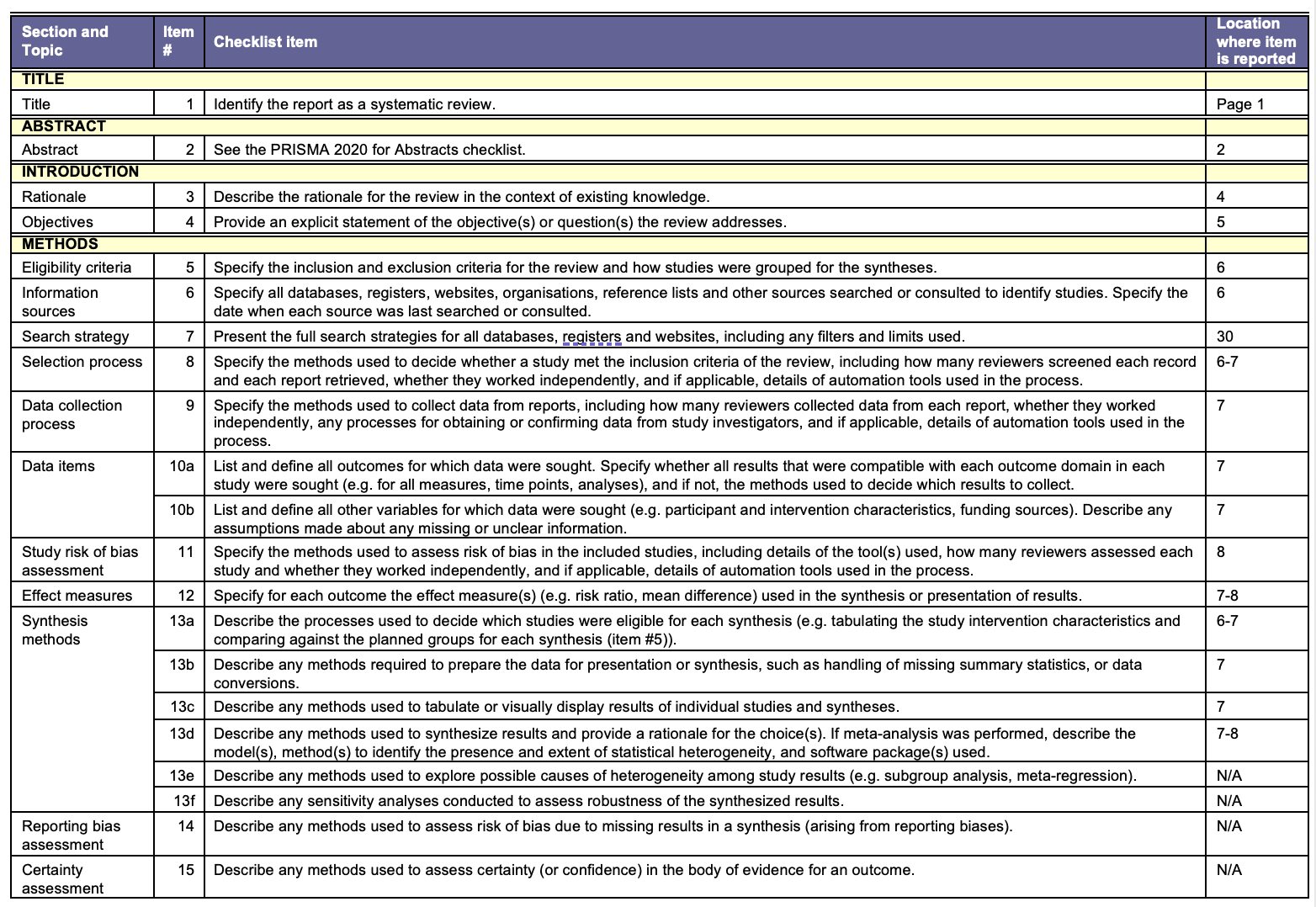

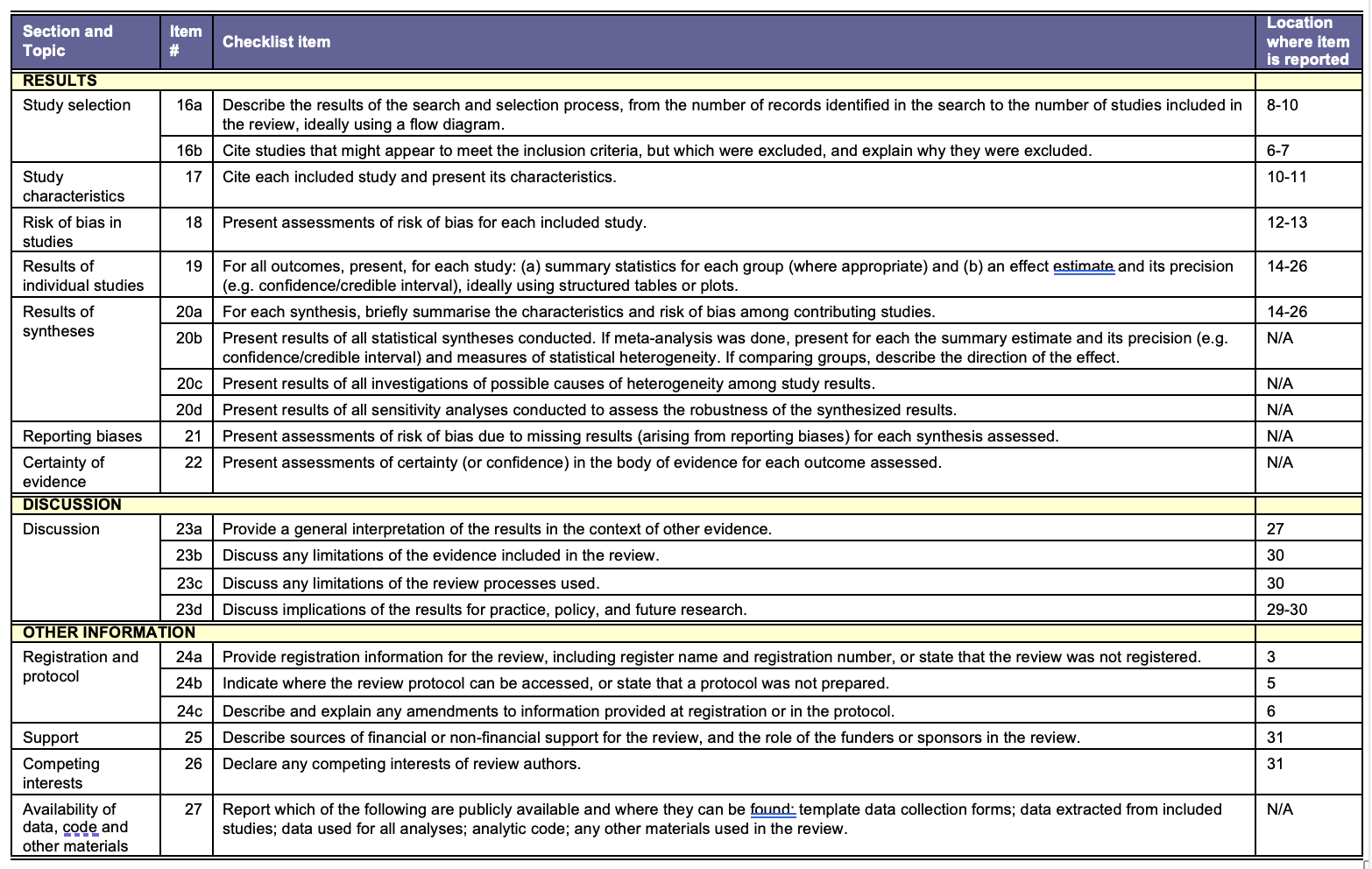

Supplement: S1 Checklist — (DOCX) [file pdig.0000772.s003.docx]
