# Supplementary material for: The behavioural and cognitive impacts of digital educational interventions in the emergency department: A systematic review
Source: PLOS Digit Health. 2025 Mar 26;4(3):e0000772. doi: 10.1371/journal.pdig.0000772 (PMC11942422; doi:10.1371/journal.pdig.0000772)
Supplement: Attachment — Submitted filename: Response to Reviewers (1).docx [file pdig.0000772.s006.docx]

PLOS Digital Health Rebuttal Letter – Paper PDIG-D-24-00184

Date: Jan 8, 2025

Dear Editors and Reviewers of PLOS Digital Health,

We greatly appreciate your thoughtful feedback and the time granted to complete these revisions. We have addressed each reviewer comment to the best of our abilities. Please see below a description of how we responded to all reviewer comments, highlighted in yellow.

Kind regards,

The authors

6. Review Comments to the Author

Please use the space provided to explain your answers to the questions above. You may also include additional comments for the author, including concerns about dual publication, research ethics, or publication ethics. (Please upload your review as an attachment if it exceeds 20,000 characters)

Reviewer #1: Thanks for addressing most of the previous queries. There are a few more things to consider/ address in the discussion section:

1. I will note that there is a paragraph in the discussion that seems underdeveloped. This is the paragraph starting with: "Interestingly, digital educational interventions seem less effective in terms of behavioural outcomes..."
-Is this supposed to say that "educational interventions are not effective for changing behavioural outcomes"?
-I find the message in this paragraph not clear - are you trying to say that health seeking behaviours are not the same as literacy comprehension?

The paragraph was edited and added to for clarity and further conceptual development.

2. The paragraph following that discusses age-differences across studies - there was a claim about assuming parents are the ones involved, please cite the papers or other literature to amplify this point. I don't doubt this, but I do find that there needs to be some solid evidence to make this claim especially when we want to consider possibly using digital interventions in the pediatric population. The papers with parents participating on behalf of their children were cited.
-"The success of interventions in studies using parents and caregivers may be explained by higher digital literacy and lower digital exclusion as compared to older populations..." I think another point of interest is to describe whether the adult studies included caregivers - comparing the effectiveness of digital interventions between caregivers and older adults is not a "fair comparison". Perhaps a discussion about whether there are studies that included caregivers for those older individuals because digital interventions are becoming a popular tool for involving all people in the circle of care. This discussion was clarified and expanded upon with additional sources.

3. Why do you think videos are the most common/ effective modality? Is it the visual aspects? The demonstration in the video, the pausing between segments etc? Think this thought can be further developed. Hypotheses as to why videos were the most effective modality were proposed with supporting literature.
