# Supplementary material for: The behavioural and cognitive impacts of digital educational interventions in the emergency department: A systematic review
Source: PLOS Digit Health. 2025 Mar 26;4(3):e0000772. doi: 10.1371/journal.pdig.0000772 (PMC11942422; doi:10.1371/journal.pdig.0000772)
Supplement: Attachment — Submitted filename: Response to Reviewers.docx [file pdig.0000772.s005.docx]

PLOS Digital Health Rebuttal Letter – Paper PDIG-D-24-00184

Date: July 25, 2024

Dear Editors and Reviewers of PLOS Digital Health,

Thank you sincerely for your helpful feedback on our paper, and for granting us extra time to execute the required work. We have addressed all the reviewer’s comments to the best of our abilities, including performing a new search for our systematic review that includes papers published within the last two years. Please see below the manner in which we responded to all reviewer comments, highlighted in yellow.

Kind regards,

The authors

Please include the following items when submitting your revised manuscript:

*	A rebuttal letter that responds to each point raised by the editor and reviewer(s). You should upload this letter as a separate file labeled 'Response to Reviewers'.

Included

*	A marked-up copy of your manuscript that highlights changes made to the original version. You should upload this as a separate file labeled 'Revised Manuscript with Track Changes'.

Included

*	An unmarked version of your revised paper without tracked changes. You should upload this as a separate file labeled 'Manuscript'.

Included

If you would like to make changes to your financial disclosure, please include your updated statement in your cover letter. Guidelines for resubmitting your figure files are available below the reviewer comments at the end of this letter.

We look forward to receiving your revised manuscript.

Kind regards,

Jennifer N Avari Silva, MD

Section Editor

PLOS Digital Health

Journal Requirements:

Additional Editor Comments (if provided):

Please see above comments from R1.

[Note: HTML markup is below. Please do not edit.]

Reviewers' comments:

Reviewer's Responses to Questions

<font color="black"><b>Comments to the Author</b><br><br>

1. Does this manuscript meet PLOS Digital Health’s <a href="https://journals.plos.org/digitalhealth/s/journal-information#loc-criteria-for-publication" target="_blank">publication criteria</a>? Is the manuscript technically sound, and do the data support the conclusions? The manuscript must describe methodologically and ethically rigorous research with conclusions that are appropriately drawn based on the data presented.</font>

Reviewer #1: Yes

--------------------

<font color="black">2. Has the statistical analysis been performed appropriately and rigorously?</font>

Reviewer #1: N/A

--------------------

<font color = "black">3. Have the authors made all data underlying the findings in their manuscript fully available (please refer to the Data Availability Statement at the start of the manuscript PDF file)?<br><br> The <a href="https://journals.plos.org/digitalhealth/s/data-availability" target="_blank">PLOS Data policy</a> requires authors to make all data underlying the findings described in their manuscript fully available without restriction, with rare exception. The data should be provided as part of the manuscript or its supporting information, or deposited to a public repository. For example, in addition to summary statistics, the data points behind means, medians and variance measures should be available. If there are restrictions on publicly sharing data—e.g. participant privacy or use of data from a third party—those must be specified.</font>

Reviewer #1: Yes

--------------------

<font color = "black">4. Is the manuscript presented in an intelligible fashion and written in standard English?<br/><br/>PLOS Digital Health does not copyedit accepted manuscripts, so the language in submitted articles must be clear, correct, and unambiguous. Any typographical or grammatical errors should be corrected at revision, so please note any specific errors here.</font>

Reviewer #1: Yes

--------------------

<font color = "black">5. Review Comments to the Author<br/><br/>Please use the space provided to explain your answers to the questions above. You may also include additional comments for the author, including concerns about dual publication, research ethics, or publication ethics. (Please upload your review as an attachment if it exceeds 20,000 characters)</font>

Reviewer #1: Thank you for giving me the opportunity to review this paper. Overall, digital interventions are a hot topic and appreciate more studies that are looking at digital interventions in discrete professions/ areas. I will note that "digital interventions" is a very broad topic, but can appreciate the difficulty or lack of evidence to focus only on one modality at this point in time. My major concerns are: 1) the search is outdated, the last time a search was run based on the manuscript was Dec 2022 - this paper cannot be published until a new search is conducted; 2) please change the language around "non-significant improvement" - if there is no statistical significance, there is NO significance, there is no improvement - writing "non-significant improvements" is misleading to readers, especially for those who are not familiar to research, or authors can use "non-significant changes", but definitely not "non-significant success/ unsuccess"

1) We have performed an updated search of the paper by replicating the previous search for the time period between December 2022 and June 2022. The eligible articles from the second search have been integrated into the paper accordingly.
2) We have changed the categorization of results across the paper to include only the categories “significant improvement”, “non-significant change”, or “significant worsening”

In the introduction:

-authors mentioned in line 70 that "discharge instructions has been found to be limited to some degree 78% of the time.." what kind of limitations are we talking about? Is it staffing limits? Resources? Not quite clear because the latter half of the sentence is talking about patients' literacy;

The language was changed to clarify the quoted result

-authors mentioned that "patient comprehension of health information is low during a clinical encounter" what are you referring to? Patient's lack of understanding because of their low literacy? Being acutely ill?

The language was changed to clarify the intended message

-the beginning of paragraph 2 details how digital educational interventions can help bridge gaps... etc. but how can digital educational interventions help bridge gaps if patients have inherently low literacy level? If a patient doesn't understand English well, an English video likely isn't going to address this limitation - how can a different modality help? Also, this sentence needs references, can't make claims about how digital interventions can help and provide no citations;

Several citations were added in this paragraph and wording was changed to clarify how digital educational interventions may help bridge gaps in understanding related to health literacy that are present with traditional methods

Methods: Greatest concern is this is supposed a systematic review and the latest search was December 2022 - there must be an updated search done within this year before this paper can be published;

An updated search was performed for papers published within this year

-Please provide full details of the library, i.e., Health sciences librarian of Faculty XXX of XX University;

The librarian’s title and university were added – the librarian does not belong to a specific faculty.

Exclusion criteria:

-mentioned "used physicians as study population" - not sure I understand this exclusion criteria, thought this study was focused on patient/ caregivers, so why would physicians be the only discipline excluded? Or did authors mean that anyone else BESIDES from patients/ caregivers, are excluded?

Wording was changed to clarify exclusion criteria for the study population

-#3 digital interventions that did not include education - so are we excluded apps that were meant for physical activity tracking for example? Like would something like Fitbit + app be excluded? please be clear;

Wording was changed to clarify exclusion criteria for the intervention

Results:

-Table 2, what is value? Is this supposed to be study #? i.e., n=?

Changed the column title to be more descriptive

-Table 4, like the illustrations in this table, but do not like the 5 different categories used - please change it to successful, unsuccessful or no changes - to my point above that if there is no statistical significance, it is not significant! It's confusing for readers, so stick to the 3 categories, the discussion is where you can talk about how there are studies that show positive/ negative trends that did not reach statistical significance, this would strengthen your discussion points;

The categories for all tables were changed to the recommended three categories, and a point was added to the discussion about trends without statistical significance

-Tables 5/6 - this goes back to your categories, keep it successful, unsuccessful, no change - the 5 categories you have now is extremely difficult to follow (confusing) - it is also not clear to me why certain rows / columns are bolded and others are not;

The categories for all tables were changed to the recommended three categories, and text was added to the figure legend to explain the bolding

Discussion:

-to reflect the points above, please change the language of "non-significantly successful" it is "not significant, but there may be potential due to the noted trend" - agree that these may be present due to the lack of high n in these studies (lines 348-350);

The language of the discussion was changed to reflect the use of the three suggested categories

-since you added data for pediatrics and adults, there needs to be some discussion about this - were there differences in the # of statistical significantly successful in adults vs children studies? How about the ones that had both populations? there are also age-related factors that may explain the success/ unsuccessful rates, which the authors did not discuss at all. One of the key barrier/ limitation we talk about in digital technology for patients is the "digital divide" - will it actually help those who are older and may not be interested? also what are the age range in these studies that had children?

This section of the discussion was further developed to answer the questions and suggestions posed by the reviewer.

--------------------

<font color = "black">6. PLOS authors have the option to publish the peer review history of their article (<a href="https://journals.plos.org/digitalhealth/s/editorial-and-peer-review-process#loc-peer-review-history" target="_blank">what does this mean?</a>). If published, this will include your full peer review and any attached files.<br><br>

<b>Do you want your identity to be public for this peer review?</b> If you choose “no”, your identity will remain anonymous but your review may still be made public.<br><br>

For information about this choice, including consent withdrawal, please see our <a href="https://www.plos.org/privacy-policy" target="_blank">Privacy Policy</a>.</font>

Reviewer #1: No
